# Poisson-noise weighted filter for time-of-flight positron emission tomography

**DOI:** 10.1186/s42492-020-00048-8

**Published:** 2020-04-29

**Authors:** Gengsheng L. Zeng, Li Lv, Qiu Huang

**Affiliations:** 1 Department of Engineering, Utah Valley University, 800 West University Parkway, Orem, UT 84058 USA; 2 Department of Radiology and Imaging Sciences, University of Utah, 729 Arapeen Drive, Salt Lake City, UT 84108 USA; 3 School of Biomedical Engineering, Shanghai Jiaotong University, Shanghai, 200240 China

**Keywords:** Positron emission tomography, Time-of-flight, Analytic reconstruction, Noise control, Nonstationary filter

## Abstract

Image reconstruction for list-mode time-of-flight (TOF) positron emission tomography (PET) can be achieved by analytic algorithms. The backprojection filtering (BPF) algorithm is an efficient algorithm for this task. The conventional noise control method for analytic image reconstruction is the use of a stationary lowpass filter, which does not model the Poisson noise properly. This study proposes a nonstationary filter for Poisson noise control. The filter is implemented in the spatial domain in a form similar to convolution.

## Introduction

Analytic image reconstruction methods for list-mode time-of-flight (TOF) positron emission tomography (PET) have been developed over the years [1–4]. One of the advantages of using TOF technology is its ability to reduce the image noise. If an iterative algorithm is used to reconstruct the image, the Poisson noise model is readily implemented as a weighting function for the projections [2]. For analytic reconstruction, the conventional noise control method is the application of a lowpass filter [5]. A lowpass filter is normally shift-invariant and can be implemented as convolution in the spatial domain or as multiplication in the Fourier domain [6]. The conventional lowpass filters thus are unable to model the Poisson noise accurately, because Poisson noise in an image is not stationary.

The goal of this study is to develop a nonstationary (i.e., shift variant) filter for Poisson noise control in analytic TOF PET image reconstruction. The filter can be two-dimensional (2D) or three-dimensional (3D). The filter developed in this study is specially targeted towards the backprojection filtering (BPF) algorithm used for TOF PET reconstruction [3, 4]. In the BPF algorithm, each event is first backprojected into the image domain. This backprojection can add a value only to one point in the image domain or add a one-dimensional (1D) Gaussian function along the line-of-response (LOR), where the point or the peak of the 1D Gaussian is at the location determined by the TOF information.

It is understood that the TOF information is not accurate and has some uncertainty. This uncertainty can be modeled as a 1D Gaussian function with a standard deviation of σ_1_. The TOF backprojection puts a different 1D Gaussian function along the LOR, and this backprojection Gaussian function can be characterized by its standard deviation σ_2_. As pointed out in refs. [3, 4], σ_2_ and σ_1_ are independent, and the user has freedom to choose σ_2_. The combined effect of σ_1_ and σ_2_ will be inverted by the tomographic filter, which depends on the sum of σ_1_ + σ_2_. Using today’s typical TOF uncertainty value, the tomographic filter can be approximated by a ramp filter (also known as the ρ filter).

Let σ_2_ = 0, which means that the TOF backprojector adds each event to a point in the image domain. The location of the point may not be the true location where the positron/electron annihilation happens due to the TOF information uncertainty. Clearly, the TOF backprojected image contains the accumulation of the photon counts at each pixel, and the noise follows the Poisson distribution. This is also true for σ_2_ > 0. The mean value and the variance are the same for a Poisson distributed random variable.

Methods Section develops a nonstationary filter for the Poisson noise. Some 2D computer simulations are presented in Results Section. The following is Discussion Section, and Conclusions Section concludes the study. The Matlab® code for the proposed filter is presented in the Appendix.

## Methods

This section develops a nonstationary filter for the 2D case. The 3D case is similar and can be readily obtained without any difficulties. Let *f* be the unfiltered image and *h* be the filter kernel. The conventional linear shift-invariant filter can be expressed as a convolution integral

1 $$ g\left(x,y\right)=\iint h\left(x-u,y-v\right)f\left(u,v\right)\  dudv $$

where *g*(*x*, *y*) is the filtered image. In Eq. (1), the kernel *h* is shift-invariant. In other words, the shift-invariant filter blurs the image *f* with the same kernel *h* everywhere. If the kernel *h* varies from location to location, Eq. (1) can be modified to

2 $$ g\left(x,y\right)=\iint h\left(u,v;x,y\right)f\left(u,v\right)\  dudv $$

Equation (2) is no longer a convolution. The calculation complexity of Eq. (2) is almost the same as the complexity of Eq. (1), except that in Eq. (2) the kernel *h* must be evaluated differently for different locations (*x*, *y*).

In this study, we assume the filter kernel *h* to be a 2D Gaussian function with a standard deviation σ(*x*, *y*). This 2D Gaussian filter with σ (*x*, *y*) is different form the 1D TOF uncertainty Gaussian function with σ_1_ and is also different from the 1D TOF backprojection Gaussian function with σ_2_.

Let us further assume that *f* is a TOF backprojected image using σ_2_ = 0. The image *f* contains Poisson noise. Each pixel of *f* is treated as a Poisson distributed random variable, and hence the variance of a pixel in *f* is the mean value of the pixel. In practice, the mean value of each pixel of *f*  is unknown, because we only have one noise realization. We thus assume that the mean value is the one realization of the image intensity of *f*.

Our strategy is to use a large kernel size of *h* if the corresponding *f* value is large and a small kernel size if the corresponding *f* value is small. The kernel size σ (*x*, *y*) is thus a monotonic function of the image pixel *f* (*x*, *y*). In this study, we empirically propose

3 $$ \sigma \left(x,y\right)=a\times f{\left(x,y\right)}^b+c $$

where σ (*x*, *y*) is the standard deviation value for the multidimensional Gaussian kernel *h* at pixel (*x*, *y*). In Eq. (3), *a*, *b* and *c* are user-selected parameters.

As a special case of *a* = 0, *h* has a constant σ (*x*, *y*), the filter is shift invariant, and Eq. (2) reduces to Eq. (1).

## Results

**Fig. 1:**
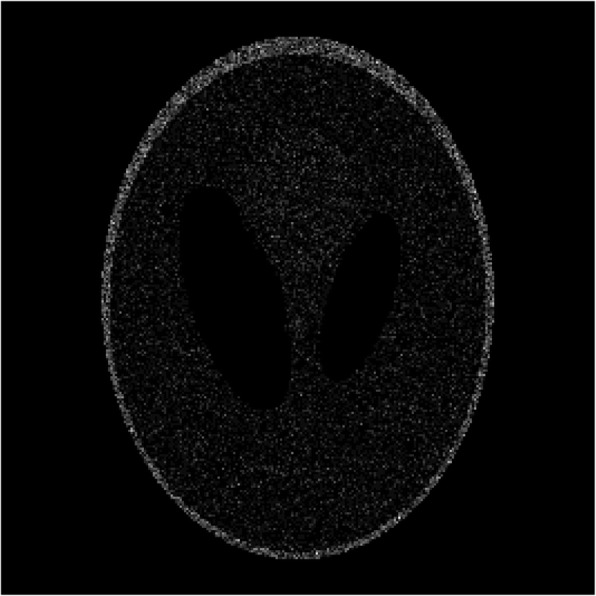
The original noisy image, where the Shepp-Logan phantom is corrupted by the Poisson noise. The RMSE is 1.0759

**Fig. 2:**
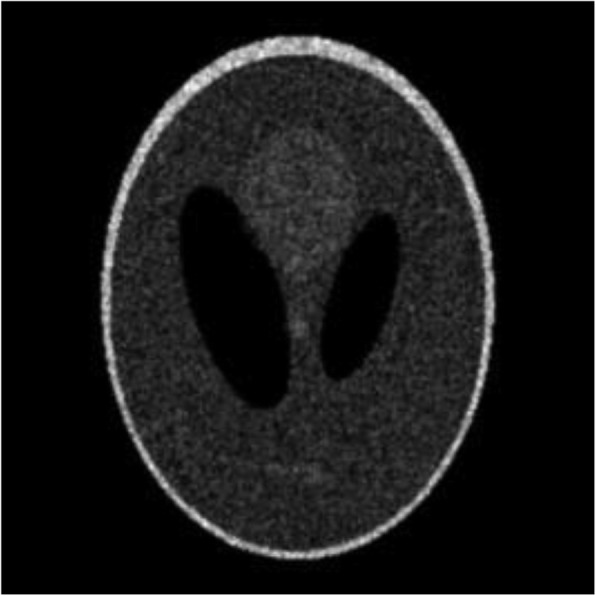
The filtered image, where a shift-invariant filter is applied with *a* = 0 and *c* = 0.73 in Eq. (3). The RMSE is 0.6093

**Fig. 3:**
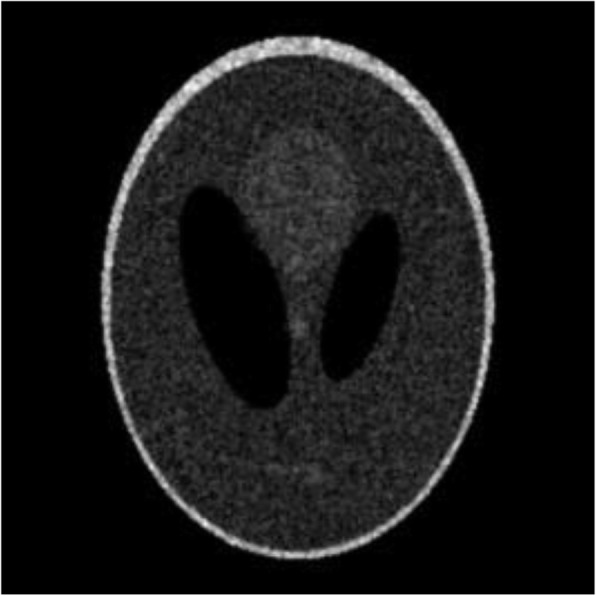
The filtered image, where a nonstationary filter is applied with *a* = 0.175, *b* = 0.01 and *c* = 0.6 in Eq. (3). The RMSE is 0.6010

Computer simulations were carried out using the Shepp-Logan phantom [7]. The image size was 256 × 256. This Shepp-Logan image was assumed to be the TOF backprojected image. Poisson noise was incorporated into the image. The original noisy image was shown in Fig. 1. Two filtered images are shown in Figs. 2 and 3, respectively. In Fig. 2, a shift-invariant filter was used with *a* = 0 and *c* = 0.73, where *c* = 0.73 was the optimal value in terms of the root-mean-square-error (RMSE) when *a* was set to 0. In Fig. 3, a nonstationary filter was used with *a* = 0.175, *b* = 0.01, and *c* = 0.6.

The RMSE was calculated for each of these 3 images compared to the true image. The RMSE’s for Figs. 1, 2 and 3 are 1.0759, 0.6093, and 0.6010, respectively. The RMSE has been reduced by switching the stationary filter to the nonstationary filter.

## Discussion

A unique feature of the proposed denoising algorithm is its non-stationarity. The non-stationary noise exists in PET and in other imaging modalities. Similar to PET, the noise in single photon emission computed tomography (CT) also contains Poisson noise [8]. In x-ray CT, the line-integral data is the logarithm of the Poisson noise corrupted transmission measurements, and the noise variance is spatially varying. Its noise variance can be approximated by an exponential function of the measurements [8]. Speckling noise in medical ultrasound images is nonstationary with a multiplicative noise model [9]. The denoising algorithm developed in this study can be readily modified to these applications. Of course, if the image noise is additive and stationary, there is no need to use the proposed algorithm. A common bandpass filter or lowpass filter may be sufficient for the denoising purpose. The stationary filters are much faster than the algorithm developed in this study. An example of such an imaging modality is photoacoustic imaging [10–12], which has an additive noise model and the noise variance is approximately stationary. Other advantages of photoacoustic imaging are that it does not use ionizing radiations and it has very high spatial resolution.

Nowadays deep learning is the most active research area, and much work has been reported in PET denoising [13–19]. Deep learning denoising is effective and has better results than traditional methods. One popular approach is the post-processing neural network that improves the signal-to-noise ratio in the raw reconstruction, which is obtained by using the filtered backprojection algorithm or the ordered-subset expectation-maximization algorithm. As a result, the low-dose PET images may have the image quality of the regular-dose PET. Instead of post-processing, another approach is to use the neural network for image reconstruction. Deep learning methods require data pairs to train the network. Good results are based on whether the current data is closely relevant to the training data sets. Our proposed algorithm is not a machine learning approach and does not require any data to train. The requirement of using our developed algorithm is that we need to know how the image noise variance dependency on the mean image. This means image is replaced by the raw image in practice. For the BPF PET image reconstruction, the raw image is the backprojected image (before the tomographic filter is applied).

## Conclusions

This study develops a nonstationary filter for the list-mode TOF PET’s BPF image reconstruction algorithm. The BPF algorithm consists of two steps: TOF backprojection and tomographic filtering. The proposed denoising filter is applied between these two steps. In other words, the nonstationary filter is applied to the TOF backprojected image.

The filter can be 2D or 3D, and its kernel width depends linearly on the intensity of the TOF backprojected image according to Eq. (3). The user needs to select parameters *a* and *b* according to the noise level and experiences. When *a* = 0, the filter degenerates to a shift-invariant filter.

## Data Availability

Not applicable.

## Appendix

### Matlab Code for the proposed nonstationary filter

% Larry, 12/17/2019% TOF PET Post Filter.

hsize = 11; % kernel size (odd int).

h2 = floor (hsize/2); % half kernel size.

isize0 = 256; % image size.

% Try (1) a = 0, c = 0.73 (RMSE = 0.6093); (2) a = 0.175, b = 0.01, c = 0.6% (RMSE = 0.6010); Original noisy image RMSE = 1.0759.

a = 0.;

b = 0.01;

c = 0.73;

rng (1); % random number seeding.

ph 0 = 10 * phantom (isize0); % Sepp-Logan phantom.

ph = padarray (ph 0, [h2 h2]); % Pad zeros.

ph = imnoise (ph*1e-12,’ poisson’) * 1e12; % Add Poisson noise.

im_out = zeros (size (ph)); % Output initialization.

Figure (2), imshow (ph, []), title (‘noisy image’).

for i = h2 + 1:isize0 + h2.

for j = h2 + 1:isize0 + h2.

temp = ph (i-h2:i + h2, j-h2:j + h2); % image patch.

sigma = a * (ph (i, j)).^b + c; % kernel width.

h = fspecial (‘gaussian’, hsize, sigma); % Filter kernel.

im_out (i, j) = dot (h(:), temp (:)); % inner product.

end

end

Figure (4), imshow (im_out, []), title (‘filtered image’).

I = im_out;

imax = max (I (:));

imin = min(I (:));

I = (I - imin)/(imax - imin);

% imwrite (I,’ a175b01c61.tif’) % RMSE 0.6010.

imwrite (I,’ a0c73.tif’) % RMSE 0.6093.

I = ph;

imax = max (I (:));

imin = min (I (:));

I = (I - imin)/(imax - imin);

imwrite (I,’ noisyImage.tif’).

ph 1 = padarray (ph 0, [h2 h2]);

ph 1 = (ph 1-im_out).^2;

MSE = sqrt (sum(ph 1(:))/ length (ph 1(:))) % RMSE.

ph 1 = padarray (ph 0, [h2 h2]);

ph 1 = (ph-ph 1).^2;

noisyMSE = sqrt (sum (ph 1(:))/ length (ph 1(:))) % RMSE.

## Abbreviations

CT: Computed tomography
PET: Positron emission tomography
TOF: Time-of-flight
LOR: Line-of-response
1D: One-dimensional
2D: Two-dimensional
3D: Three-dimensional
BPF: Backprojection filtering
RMSE: Root-mean-square-error
